# Quantitative trait linkage analysis of longitudinal change in body weight

**DOI:** 10.1186/1471-2156-4-S1-S7

**Published:** 2003-12-31

**Authors:** Astrid Golla, Konstantin Strauch, Johannes Dietter, Max P Baur

**Affiliations:** 1 Institut für Medizinische Biometrie, Informatik und Epidemiologie, Sigmund-Freud-Strasse 25, 53105 Bonn, Universität Bonn, Bonn, Germany

**Keywords:** Quantitative trait linkage analysis, longitudinal change, weight change, regression slope, metabolic syndrome

## Abstract

One of the great strengths of the Framingham Heart Study data, provided for the Genetic Analysis Workshop 13, is the long-term survey of phenotypic data. We used this unique data to create new phenotypes representing the pattern of longitudinal change of the provided phenotypes, especially systolic blood pressure and body weight. We performed a linear regression of body weight and systolic blood pressure on age and took the slopes as new phenotypes for quantitative trait linkage analysis using the SOLAR package. There was no evidence for heritability of systolic blood pressure change. Heritability was estimated as 0.15 for adult life "body weight change", measured as the regression slope, and "body weight gain" (including only individuals with a positive regression slope), and as 0.22 for body weight "change up to 50" (regression slope of weight on age up to an age of 50). With multipoint analysis, two regions on the long arm of chromosome 8 showed the highest LOD scores of 1.6 at 152 cM for "body weight change" and of >1.9 around location 102 cM for "body weight gain" and "change up to 50". The latter two LOD scores almost reach the threshold for suggestive linkage. We conclude that the chromosome 8 region may harbor a gene acting on long-term body weight regulation, thereby contributing to the development of the metabolic syndrome.

## Background

One of the special qualities of the Framingham Heart Study data, provided by the Genetic Analysis Workshop 13 (GAW13), is the long-term survey of phenotypic data. The quantitative phenotypes systolic blood pressure, body height, body weight, fasting serum glucose, total plasma cholesterol, high density lipoprotein cholesterol, and triglycerides were measured repeatedly over a period of 40 years in the first generation of study participants and over a period of 20 years in their offspring. We used this unique data to create new phenotypes representing the pattern of longitudinal change of the provided phenotypes, assuming that not only the measured traits, but also their pattern of longitudinal change, could be genetically determined. We chose the phenotypes systolic blood pressure and body weight because of their known tendency to increase during adult lifespan, and because of their most complete longitudinal survey by repeated measurements at each examination during the study. Our own previous analyses of the population sample of Framingham Cohort 1 and 2 showed that body weight and systolic blood pressure rise linearly with age, but the gradient is steeper when the regression is performed only up to an age of 50 compared with the regression on all available measurements. Another group participating in Genetic Analysis Workshop 13 (GAW13) analyzed the changes of body mass index with age (BMI = weight/height^2^, which corresponds to weight change, because there is almost no change of height during adult life). They found a linear gain-phase up to a maximum value (at about age 53 on average) and a decline-phase later in life [1]. According to this we analyzed "body weight change" during whole life, "body weight gain" (using phenotypes of individuals with a positive gain of weight only), and body weight "change up to 50", the regression slope of weight on age up to an age of 50.

## Materials and Methods

The Framingham Heart Study data were provided as Problem 1 of GAW13. The data consisted of family structure information, genotypic information (genome scan), and long-term phenotypic information for 1702 subjects from 330 families. Phenotypic information was provided over a period of 40 years for the parent generation and 20 years for the offspring generation. Phenotypic information consisted of values of repeated measurements of systolic blood pressure, height, weight, lipids (total plasma cholesterol, high density lipoprotein cholesterol, triglycerides), and fasting plasma glucose. We chose the phenotypes systolic blood pressure and body weight for our analysis.

### Statistical analysis

Linear regression was perfomed using SAS [2] for the values of systolic blood pressure and body weight, at each time of examination, on age, for each individual separately. To obtain the values for adult life only, individuals younger than 18 years at the first examination were excluded. We omitted the values of these individuals instead of using the examinations with age 18 as a starting point because of lower reliability of the regression slopes (based on four time points at most and often less due to younger age and missing values) and because of their relatively low contribution due to small frequency (only 6% of individuals of Cohort 2 and none of Cohort 1 were younger than 18 years at their first examination). For systolic blood pressure, measurements were omitted if high blood pressure treatment was stated at this examination or any previous examination. The slope estimates were obtained for all individuals and used as a new phenotype providing a measure of the adult life change of systolic blood pressure and body weight. Further analyses of body weight change were conducted with regression slope up to an age of 50 as the phenotype "change up to 50" and with only those individuals who showed a positive weight slope (≥ 0.01), representing the phenotype adult life "body weight gain". Heritability estimates for the new phenotypes were obtained using variance-component methodology as implemented in the SOLAR package [3]. Heritability estimates were done analyzing the covariates sex, age at first examination and weight at first examination in addition. Only age at first examination contributed to the variance of the three phenotypes, explaining about 12% of the variance of "body weight change", 6% of the variance of "body weight gain", and 9% of the variance of "change up to 50". Because further examinations were at the same regular intervals for all individuals of the two cohorts, age at first examination represents age-dependence during the whole study. Two-point and multipoint quantitative trait linkage analyses were conducted on the regression slope estimates for adult life "body weight change", "body weight gain", and "change up to 50", using the SOLAR package. In the case of "body weight gain", we included age at first examination as a covariate. The information content of chromosomal regions with positive LOD scores was obtained using GENEHUNTER [4].

## Results

**Figure 1:**
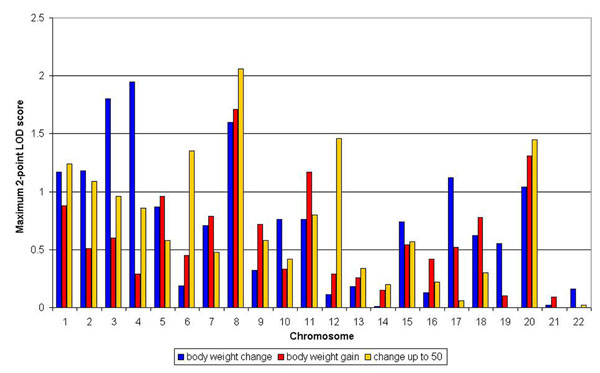
**Maximum two-point LOD scores for body weight change phenotypes for the 22 autosomes. **Depicted are "body weight change" (blue), "body weight gain" (red), and "change up to 50" (yellow).

**Figure 2:**
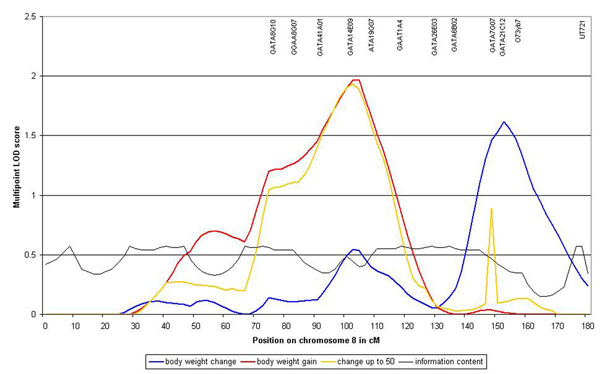
**Multipoint LOD scores for body weight change phenotypes on chromosome 8. **Depicted are "body weight change" (blue), "body weight gain" (including the covariate age at first examination) (red), and "change up to 50" (yellow).

The estimated heritability for adult life "blood pressure change" was 0.002 (not significant). For adult life "body weight change" a heritability of 0.15 (highly significant, *p *< 0.0001) was estimated. The highest two-point LOD scores for "body weight change" for each of the 22 autosomal chromosomes are presented in Figure 1. The highest LOD scores were 1.95 at marker GATA8A05 (D4S1629) on chromosome 4, 1.8 at marker GATA6G12 (D3S2398) on chromosome 3, and 1.6 at marker GATA7G07 (D8S1179) on chromosome 8. Information content in these chromosomal regions varied mostly from 0.33 to 0.55 and did not exceed 0.60. All other two-point LOD scores were below 1.5 (Figure 1, blue bars). Inclusion of age at first examination in the heritability model resulted in low two-point LOD scores (<1.6) over all chromosomes and a heritability model without covariates was used in multipoint analysis. Multipoint LOD scores diminished with respect to the two-point LOD scores for the regions on chromosome 3 and 4 with maximum values of 1.37 (location 207 cM on chromosome 3) and 1.10 (location 181 cM on chromosome 4). These lower values were due to low LOD scores of the flanking markers (between 0.06 and 0.28 for chromosome 3 and 0.06 to 0.31 for chromosome 4). Only the region on chromosome 8 showed stable LOD scores (maximum multipoint LOD score 1.61 at position 152 cM) (Figure 2, blue line). For adult life "body weight gain", which included phenotypic information only for those individuals with positive slope estimates, heritability was estimated as 0.15 (highly significant, *p *< 0.0001). Age at first examination was included as a covariate in the heritability model with an estimated attributable proportion of variance of 0.06. The highest two-point LOD score for "body weight gain" was 1.7 at GATA14E09 (D8S2324) on chromosome 8 with all other two-point LOD scores below 1.5 (including markers on chromosomes 3 and 4, where LOD scores for the phenotype "body weight change" were higher) (Figure 1, red bars). The maximum multipoint LOD score was 1.97 at location 103 cM (near GATA14E09) on chromosome 8 (Figure 2, red line). For "change up to 50", the regression slope of weight on age up to an age of 50, a heritability of 0.22 (highly significant, *p *< 0.0001) was estimated. The highest two-point LOD scores were on chromosome 8, 1.85 at GATA8G10 (D8S1110) and 2.06 at GATA14E09 (D8S2324), the latter marker being the position of the highest two-point LOD score of "body weight gain", too. All other two-point LOD scores were below 1.5 (Figure 1, yellow bars), as were all two-point LOD scores when age at first examination was included as a covariate in the heritability model. The maximum multipoint LOD score for "change up 50" (using a heritability model without covariates) was 1.93 at 102 cM of chromosome 8 with a LOD score peak and curve almost identical to that of "body weight gain" (Figure 2, yellow line). The two regions of maximum LOD scores for "body weight change" and "body weight gain"/ "change up to 50" are about 50 cM apart on the long arm of chromosome 8 (Figure 2).

## Discussion

Adult life increase of systolic blood pressure and of body weight are known phenomena, at least in western lifestyle populations. Environmental factors like lack of exercise and high food intake are major factors influencing both of them. Yet little is known as to whether heritable factors may contribute to the pattern of change of blood pressure and body weight during lifetime. Our analysis using regression slopes as a measure for lifetime changes showed no evidence of a heritable basis of blood pressure change, but we found heritabilities of 0.15 for "body weight change" and "body weight gain" and of 0.22 for body weight "change up to 50". We identified two regions 50 cM apart from one another on the long arm of chromosome 8 with linkage to "body weight change" (distal region) and to "body weight gain" and "change up to 50" (proximal region). Although not significant, the LOD scores for "body weight gain" of 1.97 and "change up to 50" of 1.93 at locations 102 and 103 cM on chromosome 8 almost reach the threshold of 2.2 for suggestive evidence of linkage [5]. Very large sample sizes are required for detection of a quantitative trait locus (QTL) attributable to 10% or 15% heritability of a trait with significant LOD scores. For instance, if 10% heritability is due to a single QTL, 3300–33,000 individuals (depending on family structure) are required to obtain a LOD score of 3.0 with 80% power [6]. The Framingham Heart Study sample with 1702 subjects from 330 families is very big, yet it may well be not big enough to obtain statistically significant linkage results for traits with total heritabilities of 0.15 to 0.22, even if a single locus would attribute to 0.67 or 0.50 of the heritability. We therefore think, that "almost suggestive linkage" is probably the best obtainable result for the body change traits we analyzed, and that confirmation would require analysis (or meta-analysis) of still larger sample sizes.

Interestingly, two other groups participating in the Derived Phenotypes Group of GAW13 reported related results with different (but pathophysiologically related) phenotypes. Jun et al. found statistically significant evidence of linkage between the region on chromosome 8 around marker GATA21C12 and fasting glucose levels progression (exactly the same region which we identified for "body weight change") [7]. Wilcox et al. derived four phenotypically distinct groups using correspondence analysis and clustering. One of these groups resembled atherogenic dyslipidemia and the metabolic syndrome. Besides other chromosomal regions, they found the same chromosome 8 location (two-point LOD scores above two for markers GATA7G07, UT721, GATA21C12) for their metabolic-syndrome-like phenotype as we found for "body weight change" [8]. The region on chromosome 3, where we obtained a relatively high two-point LOD score for "body weight change", contains AOMS1 (abdominal obesity-metabolic syndrome QTL1), the first known metabolic syndrome QTL [9]. These findings underscore the pathophysiological relationship between body weight change (body weight gain), blood glucose level progression, and the metabolic syndrome, and point to a long-term body weight regulating gene on chromosome 8 that may play a role in the development of the metabolic syndrome.

## Conclusion

Longitudinal data like that collected in the Framingham Heart Study provided for GAW13 enable the search not only for genes influencing the phenotypes themselves, but also for genes contributing to the pattern of change during lifetime. Using regression slope estimates as a parameter for longitudinal change of body weight, we found evidence for a long-term body weight regulating gene located on the long arm of chromosome 8. Combining our results with the findings of other GAW13 participants [7,8], we think that this gene might be involved in the development of the metabolic syndrome by influencing long-term body weight and consequently blood glucose level. Location of genes involved in long-term regulation of physiological processes can be found using phenotypes derived from longitudinal data, and exactly those long-term regulatory genes may play key roles in frequent diseases of late onset.
